# Practical Notes and Formulæ

**Published:** 1887-05

**Authors:** 


### PRACTICAL NOTES AND FORMULÆ.

**Speedy Cure for Gonorrhœa.**—In reply to your question column I will give my three-day cure for gonorrhœa:

℞. Oil sandal wood, }

Fl. ext. quillea sapo, } aa.................... ℥ iv.

M. and shake. Add

Glycerin, }

Aqua cinnamon, } aa........................ ℥ iij.

M. Sig.—Teaspoonful four times a day.

℞. Morph. sulph.............................gr. iij,
Muriate berberina..........................gr. x,
Zinci sulphas..............................gr. viij,
Bismuth. subnit............................ʒ iv,
Aqua rosa..................................℥ iv.
M. Sig: Inject a small amount after each micturition.

Keep the glans penis well covered with cloth, so as to prevent the discharge from soiling the linen. This is a very necessary precaution for a speedy cure, as matter on the clothing reinoculates and continues the disease indefinitely.—*Dr. Charles C. Edson, Chicago Medical Times*

**The Treatment of Threadworms in Children.**—Dr. Sydney Martin writes as follows to *The Practitioner:* "In many cases I have found that rhubarb in small doses brings away large numbers of worms and at the same time regulates the bowels, so that the use of injections may in most cases be dispensed with. The formula which I have found most useful is as follows, varying slightly with the age of the child:

℞. Tincture rhei.............................*m* iij,
Magnesii carbonatis........................gr. iij,
Tincturæ zingiberis........................*m* j,
Aquam, ad..................................ʒ j.

"This is to be taken twice or three times daily according to the effect on the bowels. Whether the rhubarb acts as a vermicide or simply by 'moving the worms on,' I am unable to say."—*Am. Medical Digest.*

**A journal** in Illinois recently brought suit against forty-three men who would not pay their subscriptions, and obtained judgment in each for the full amount of the claim. Of these, twenty-eight men made affidavit that they owned no more property than the law allowed them, thus preventing attachments. Then they, under the decision of the Supreme Court, were arrested for petty larceny, and bound over in the sum of $300 each. All but six gave bonds, while six went to jail. The postal law makes it larceny to take a newspaper and refuse to pay for it.—*American Medical Journal.*

**A Disinfectant Mixture for Apartments.**—A contributor to the *Union Medicale* for Dec. 16, 1886, gives the following formula:

| | | |
|---|---|---|
| ℞. | Camphor | 20 parts, |
| | Calcium hypochlorite | 50 parts, |
| | Alcohol | 50 parts, |
| | Water | 50 parts, |
| | Oil of eucalyptus | 1 part, |
| | Oil of cloves | 1 part. |

Mix in a large vessel kept cold. A few drops, on a napkin, are enough to disinfect a room.—*N. Y. Medical Journal.*

**Coughs.**—

| | | |
|---|---|---|
| ℞. | Ammonia mur., Bromide potas., aa | ʒ ij, |
| | Vinum ipecac | ʒ ij, |
| | Syr. yerba santa, Syr. prunus virginia, aa | ℥ jss. |

M. Sig.—Teaspoonful every three hours.—*Exchange.*

**For Rheumatism.**—For the special trouble, the sum of departure from health present, which we name rheumatism, I have always for some years past used the following prescription, which is both pleasant to take and the best salicylate I have ever yet found:

| | | |
|---|---|---|
| ℞. | Acid salicylic | ʒ iv, |
| | Potass. citras | ʒ vj, |
| | Ammon. carb | ʒ ij, |
| | Aqua | ℥ iij. |

Triturate in a mortar with the water until effervescence ceases, then add: syr. simp., q. s. ℥ vi. Sig.—Two teaspoonfuls every two or four hours, as the urgency of the symptoms demand. In some cases, 3 teaspoonfuls may be given at a dose. When the graver symptoms are under command continue the medicine in teaspoonful doses every three or four hours, or three to five times a day. I use the above freely and in the largest doses, even when cardiac symptoms are present.—*American Medical Journal.*

**Dr. J. Lewis Smith** suggests the following schedule for dilution of average cow's milk for infants: To the third week, three parts water; from the sixth to the fourteenth week, one-half water; from the fourteenth week to four and a half months, one-third water; from four and a half months to the sixth month, one-fourth water.—*Practice.*

**The fluid extract of jaborandi**, in the hands of Dr. G. W. Robinson, of Trindad, Cal., has proved to be a valuable local remedy in eczema. The intense itching is relieved after one or two applications.—*Practice.*

**Cimicifugia for Headache.**—The severe headache which usually ushers in the menstrual period, is relieved by cimicifuga. Congestive, nervous and rheumatic headache, and those resulting from alcoholic excesses and loss of sleep, also frequently yield to this drug. It is best given in small doses, say *m* x of the fluid extract, frequently repeated. It has been found useful in the chorea of children.—*Medical World.*

**Instantaneous Remedy for Lumbago.**—Collodion, tincture of iodine, liquid ammonia, equal parts. To be applied widely over the parts with a camel's-hair brush. This applies to accidental or *frigore* lumbago or to rheumatic pain produced by a strain or muscular exertion.—*Quarterly Compendium.*

**Atropine in Nocturnal Earaches of Children.**—In the nocturnal earaches to which so many children are subject, and from which they frequently cry a greater part of the night, the application, by dropping into the external cavity, of a few drops of a solution of atropine (2 grains to the ounce of water) will give prompt and effectul relief.—*Ibid.*

**Itch Ointment.**—The following is said by the *Rundschau* (Prag.) to be a very active remedy against itch:

| | | |
|---|---|---|
| ℞. | Flowers of sulphur | 8 parts, |
| | Liquid tar | 8 parts, |
| | Lanolin | 16 parts, |
| | Green soap | 16 parts, |
| | Powdered pumice stone | 5 parts. |

Mix.—*Quarterly Compendium.*

**Incontinence of Urine.**—Dr. Ellis, in *Diseases of Children:*

| | | |
|---|---|---|
| ℞. | Liq. strychniæ, B. P. | *m* x, |
| | Tr. belladonnæ | ℈ i-ʒ ss. |
| | Inf. cascarillæ, ad | ℥ ij. |

M. Sig.—One ʒ three times a day to a child three years old.—*Illustrated Medical Journal.*

**Cotton Root as a Uterine Hæmostatic.**—Garrigues, in the *Quarterly Bulletin*, concludes that gossypium is an ecbolic, an oxytoxic, an emmenagogue, and a corrective for menstrual pain. Besides this, it has the power of checking chronic loss of blood from the womb, no matter what may be the pathological condition. In 139 cases of eighteen different uterine complaints, this drug proved effectual in 125.—*Medical World.*

**Equal parts** of the fluid extracts of digitalis, ergot and ipecac, just enough ipecac to cause nausea, is, according to Prof. Bartholow, a good combination for *pulmonary hemorrhage.*—*Medical World.*

**Prof. Parvin** states that *pruritus vulvæ* may be sometimes due to wild hairs.

